# Metabolic Regulation of Adipose Tissue Macrophage Function in Obesity and Diabetes

**DOI:** 10.1089/ars.2017.7060

**Published:** 2018-07-20

**Authors:** Mahesh Appari, Keith M. Channon, Eileen McNeill

**Affiliations:** ^1^ Division of Cardiovascular Medicine, British Heart Foundation Centre for Research Excellence, John Radcliffe Hospital, University of Oxford, Oxford, United Kingdom.; ^2^ Wellcome Trust Centre for Human Genetics, University of Oxford, Oxford, United Kingdom.

**Keywords:** obesity, redox signaling, immunometabolism, macrophages, diabetes

## Abstract

***Significance:*** Obesity and diabetes are associated with chronic activation of inflammatory pathways that are important mechanistic links between insulin resistance (IR), type 2 diabetes (T2D), and cardiovascular disease pathogenesis. The development of these metabolic diseases is associated with changes in both the number and phenotype of adipose tissue macrophages (ATMs). Emerging lines of evidence have shown that ATMs release proinflammatory cytokines similar to classically activated M1 macrophages, which directly contribute to IR or T2D. In contrast, adipose tissue (AT) from lean healthy individuals contains macrophages with a less inflammatory M2 phenotype.

***Recent Advances:*** Recent research has shown that macrophage phenotype is linked to profound changes in macrophage cellular metabolism.

***Critical Issues:*** This review focuses on the role of macrophages in AT inflammation and obesity, and the metabolic changes in macrophage function that occur with activation that underpin their role in the pathogenesis of IR and T2D. We highlight current targets for altering macrophage metabolism from both within the field of metabolic disease and AT biology and more widely within inflammatory biology.

***Future Directions:*** As our knowledge of macrophage metabolic programming in AT builds, there will be increasing scope for targeting this aspect of macrophage biology as a therapeutic strategy in metabolic diseases. *Antioxid. Redox Signal.* 29, 297–312.

## Introduction

Obesity and type 2 diabetes (T2D) are global health problems causing substantial morbidity, early mortality, and a massive health economic burden. The World Health Organization global health observatory estimated that 10.8% of men and 14.9% of women were obese with a body mass index (BMI) of 30 or more in 2014. Human cohort studies indicate that multiple risk factors coexist in the metabolic syndrome, including obesity, insulin resistance (IR), elevated blood pressure, impaired glucose tolerance, and dyslipidemia. These patients are at increased risk for developing T2D and manifest vascular disease and increased risk for cardiovascular events and mortality (25). The metabolic consequences of adipose tissue (AT) dysfunction are more important factors in the development of cardiovascular risk in T2D patients rather than other markers of obesity such as BMI, waist circumference, and intraabdominal fat (26). Indeed, intervention studies suggest that even a modest (5%) weight loss, achieved by low-calorie diet, substantially improves metabolic functions in AT, liver and muscle insulin sensitivity, and pancreatic β-cell function (61), suggesting that changes in AT biology and metabolism are critical regulators of disease pathogenesis in obesity and T2D.

IR is central to the adverse pathology linking obesity to T2D. IR can cause T2D, in particular, when pancreatic β-cell dysfunction emerges, which can no more be compensated by adequate insulin production. Insulin produced by pancreatic β-cells lowers blood glucose through insulin receptor activation in various cell types such as adipocytes, myocytes, and hepatocytes to increase their glucose uptake. Moreover, insulin inhibits glucagon secretion by pancreatic α-cells (*via* glycogenolysis and gluconeogenesis) (4). When IR occurs, downstream insulin signaling is impaired (as evidenced by, for example, reduced Akt phosphorylation), and/or directed *via* pathways that may lead to activation of stress pathways, such as the p38 MAP kinase. IR can be caused by many factors at a cellular level such as endoplasmic reticulum (ER) stress, oxidative stress, hypoxia, dysregulation of lipid homeostasis, and mitochondrial dysfunction. However, obesity is increasingly recognized as a major contributor to IR (19).

An association between obesity and inflammation was first identified in the 1960s (56). In obese individuals, systemic inflammation is indicated by elevated circulating levels of proinflammatory cytokines such as tumor necrosis factor alpha (TNF-α) (51), and increased production of this proinflammatory cytokine by ATs (41). More recent studies have highlighted macrophages resident in ATs as a principal source for this inflammatory signature (103). This chronic metabolic inflammation (metainflammation or metaflammation) and metabolic dysfunction contribute to cardiovascular disease risk and pathogenesis in patients with obesity or T2D. Metaflammation is evident both systemically and in specific metabolic organs such as AT, liver, muscle, pancreas, brain, and gut.

Metaflammation is postulated to drive the transition toward the metabolic syndrome and T2D [reviewed in Ref. (76, 87)]. Obese individuals exhibit an elevated number of circulating leukocytes, these cells show an activated proinflammatory phenotype with enhanced expression of macrophage migration inhibitory factor (MIF), TNF-α, interleukin-6 (IL-6), and matrix metallopeptidase 9 (32). AT in these individuals has increased macrophage infiltration as body mass and adiposity increase (103, 106). Although a host of changes are seen systemically in obesity, the macrophage population resident in AT depots and other key sites of metabolic regulation appear to be key players, impacting disease progression not just through their number but also through alterations in their activation state. In this review, we discuss the role of macrophages in obesity and T2D. We focus on how cellular metabolism is altered in macrophages with different activation status, and describe how altered macrophage metabolism may impact on inflammation in diabetes and obesity.

## Adipose Tissue and Obesity

AT, composed of adipocytes (specialized mesenchymal cells), also contains several other cell types such as stromal vascular cells, endothelial cells, fibroblasts, macrophages, and other leukocytes. The three types of AT, white, brown, and beige or brite (brown-like-in-white), are classified according to the origin and function of constituent cells. Brown adipose tissue (BAT) metabolizes lipids to generate heat (thermogenesis) in response to cold and β-adrenergic stimulation (36). BAT induces nonshivering thermogenesis by expressing mitochondrial protein, uncoupling protein 1 (UCP1) [reviewed in Ref. (36)]. The white adipose tissue (WAT) depot and its expansion (*via* adipocyte hyperplasia and hypertrophy) are considered as important to obesity and IR. There are two major types of WAT namely visceral AT (VAT), present in abdominal cavity and mediastinum, and subcutaneous AT in the hypodermis. AT location and its effect on metabolic function vary because of the differential gene expression patterns of the various depots [reviewed in Ref. (10) (86)]. For example, obese (high-fat-diet [HFD]-fed) sarcolipin deficient mice had shown depot-specific increase in AT inflammation and remodeling by expressing higher levels of the proinflammatory cytokines IL-6, IL-1β, and TNF-α in the epididymal fat rather than inguinal fat (59). These gene expression patterns are well correlated with macrophage infiltration and inflammatory activation. Human cohort studies demonstrate that VAT is strongly associated with glucose intolerance and IR (84). BAT is responsible for energy expenditure in thermogenesis against cold, and short-term cold exposure increases BAT activity resulting in accelerated clearance of plasma triglyceride (TG)-rich lipoproteins (5); [reviewed in Ref. (36)].

AT stores free fatty acid (FFA) after excess food intake, wherein elevated plasma FFA enters the adipocytes, and is transformed and stored as TGs. During the fasting stage, adipocytes release FFA to balance energy status (fuelling other organs) through lipogenesis and lipolysis. FFA binds to toll-like receptor 4 (TLR4) and activates a proinflammatory response, resulting in accumulation of adipose tissue macrophages (ATMs) (18, 94). The liver is a key organ in glucose homeostasis, with adipocyte-released FFA accumulating in the liver, resulting in fatty liver disease. Elevated FFAs are associated with IR in obese patients with T2D [reviewed in Ref. (7)].

In addition to metabolic functions, AT acts as an endocrine organ. Adipocytes in AT secrete hormones (adipokines or adipocytokines) such as leptin, adiponectin, and resistin along with retinol binding protein 4 and secreted frizzled-related protein 5 (Sfrp5), which exhibit endocrine or paracrine functions and are important in maintaining energy homeostasis (16, 88, 100). Sfrp5 is an anti-inflammatory adipokine expressed mainly in the adipocytes of WAT. HFD-fed Sfrp5-deleted mice exhibited accumulation of macrophages (F4/80 and CD68), c-Jun NH2-terminal kinase (JNK) activation, an increase in proinflammatory mediators (TNF-α, IL-6, monocyte chemotactic protein-1 (MCP-1), severe glucose intolerance, and hepatic steatosis (79). Adipocytes also secrete chemokines such as MCP-1 and leukotriene B4 (Ltb4), a potent leukocyte chemoattractant and activator [reviewed in Ref. (68)]. Leukotrienes (LTs) are potent proinflammatory mediators in AT and cause IR. Inhibition of 5-lipoxygenase, an enzyme involved in the biosynthesis of LTs, in obese (HFD-fed) mice protects from IR by reduced AT macrophages and T cell infiltration (71). Adipokines exert their function on metabolic organs such as liver, skeletal muscle, pancreas, and the cardiovascular system. Adiponectin, which exerts anti-inflammatory functions, regulates uptake of FFA by muscle and WAT and decreases hepatic glucose production (7). Adiponectin also regulates glucose homeostasis and IR. In the presence of excess nutrients, the adiponectin receptor is downregulated by the activation of JNK pathway by TNF-α and IL-6 and oxidative stress (17).

## Cellular Events Associated with Metaflammation and Obesity

**FIG. 1.**
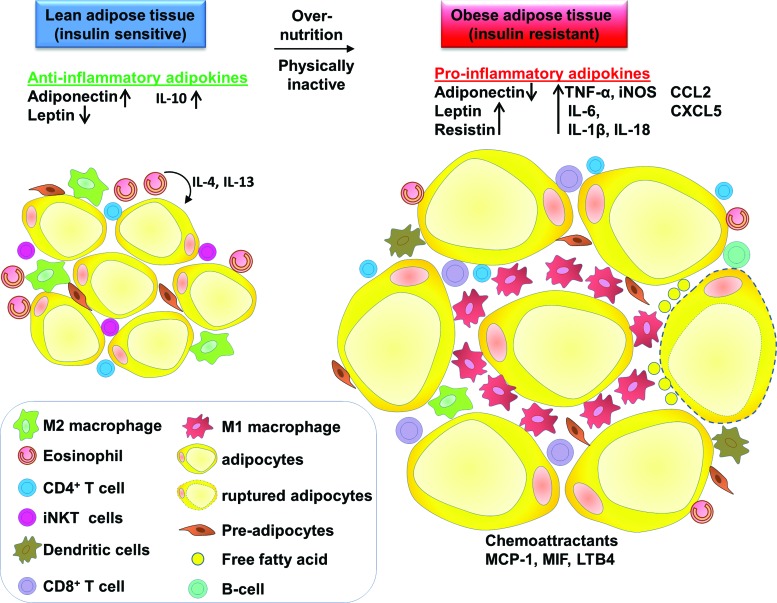
**Macrophages and other immune cells distribution and function in lean and obese AT.** In lean AT, M2 macrophages are distributed evenly throughout the tissue. Lean AT also contains CD4^+^ T cells, iNKT cells, and preadipocytes. Eosinophils secrete IL-4 and IL-13 that act on adipocytes and contribute to anti-inflammatory and insulin-sensitive state. Adipokines such as adiponectin and IL-10 are increased, whereas leptin content is decreased. In obese AT, M1 macrophages form crown-like structures around the AT. M1 macrophages increase their number by both infiltration and local proliferation. Adipocyte hypertrophy causes the rupture of adipocytes and releases FFA. M1 macrophages secrete proinflammatory cytokines. The content of other immune cells also changes with eosinophils and regulatory T cells (T_reg_) content, decreasing and dendritic cells, CD4^+^ and CD8^+^ T cell numbers increasing. The number of preadipocytes increases. Proinflammatory adipokines production such as TNF-α, IL-6, IL-1β, MCP-1, and MIF increases. AT, adipose tissue; FFA, free fatty acid; IL-6, interleukin-6; MCP-1, monocyte chemotactic protein-1; MIF, macrophage migration inhibitory factor; TNF-α, tumor necrosis factor alpha. To see this illustration in color, the reader is referred to the web version of this article at www.liebertpub.com/ars

Recent studies have revealed some of the cellular mechanisms responsible for the activation of inflammatory pathways in obesity and T2D. Obesity is associated with AT remodeling, which includes chronic low-grade activation of inflammation of AT and increased ATM infiltration, adipocyte hypertrophy, and increase in angiogenesis and extracellular matrix (27, 76). Adipocyte hypertrophy causes rupturing of adipocytes, resulting in increased local inflammatory cell accumulation, including macrophages, T cells, and altered production of adipokines (Fig. 1).

In humans, FFAs induce IR in muscle by initial inhibition of glucose transport followed by reduction in muscle glycogen synthesis and oxidation (85). FFAs can also induce IR by inhibiting glucose transport activity, which may be a consequence of decreased insulin receptor substrate-1 (IRS-1)-mediated phosphatidylinositol 3-kinase (PI3-k) activity (20). In rats, increased FFA upregulates intracellular fatty acyl-CoA and diacylglycerol (DAG) concentrations, which lead to activation of protein kinase C (PKC)-theta. PKC-theta stimulates IRS-1-mediated PI3-k activity, which increases insulin-stimulated glucose transport activity (110). FFAs from dietary fat are also important signaling molecules binding to free fatty acid receptors (FFARs), which are G protein-coupled receptors (GPCRs). Medium or long-chain fatty acids (FAs) bind and activate GPR40/FFAR1 and GPR120/FFAR4, whereas short-chain FAs activate GPR43/FFAR2. FFARs are regarded as potential therapeutic targets to reduce FFA-mediated IR (44).

**FIG. 2.**
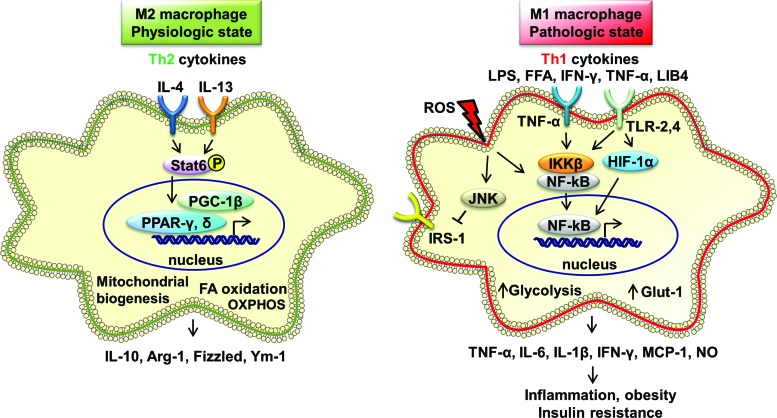
**Macrophage inflammatory pathways in physiological and pathological states of obesity and insulin resistance.** M1 macrophages are activated by inflammatory inducers, TNF-α, TLR-2, four ligands, IFN-γ, and ROS. M1 macrophages function *via* JNK and NF-κB pathways. Activated M1 macrophages release TNF-α, IL-6, IL-1β, IFN-γ, MCP-1, and NO, resulting in increased inflammation, obesity, and insulin resistance. M2 macrophages are activated by IL-4 and IL-13 that act *via* STAT6, PPAR-γ, PPAR-δ, and PGC-1β. M2 macrophages express IL-10, Arg-1, Fizzled, and Ym-1. M2 macrophages increase FA oxidation and OXPHOS. GLUT-1, glucose transporter-1; IFN-γ, interferon-gamma; IRS-1, insulin receptor substrate-1; JNK, c-Jun NH2-terminal kinase; LPS, lipopolysaccharide; NF-κB, nuclear factor-kappaB; NO, nitric oxide; OXPHOS, oxidative phosphorylation; PGC-1β, peroxisome proliferator-activated receptor gamma coactivator 1-beta; PPAR-γ, peroxisome proliferator-activated receptor-gamma; ROS, reactive oxygen species; STAT6, signal transducer and activator of transcription 6; TLR, toll-like receptor. To see this illustration in color, the reader is referred to the web version of this article at www.liebertpub.com/ars

Obesity-associated FFAs induce inflammation *via* TLR4, the receptor for lipopolysaccharide (LPS) that plays a critical role in initiating intracellular nuclear factor-kappaB (NF-κB)-mediated signaling, leading to activation of innate immunity (112) and causing IR in AT (93) (Fig. 2). In HFD-induced obese mice, lipids accumulated in the liver and caused hepatic inflammation *via* NF-κB activation, resulting in IR (14). Studies in human subjects and in mice have revealed that dietary intake of saturated fat (palm oil) induces TLR4-mediated inflammation, NF-κB activation, increases hepatic TG storage, energy metabolism, and induces IR (38).

TNF-α was identified as an early potential target in the treatment of IR in obesity (41). TNF-α released by AT may act through autocrine or paracrine functions, causing IR (3). Obesity-induced IR was improved by genetic or pharmacological inhibition of inhibitor of κB kinase, which reduces NF-κB pathway activation, thus reducing TNF-α production (57). TNF-α-treated brown adipocytes induce protein-tyrosine phosphatase 1 (PTP1B) and inhibition of PTP1B confers protection against cytokine-induced IR (21). The seminal observation of the link between TNF-α and metabolic disease has firmly focused attention on inflammation and the macrophage.

## Macrophage Phenotype in Obesity and Diabetes

**FIG. 3.**
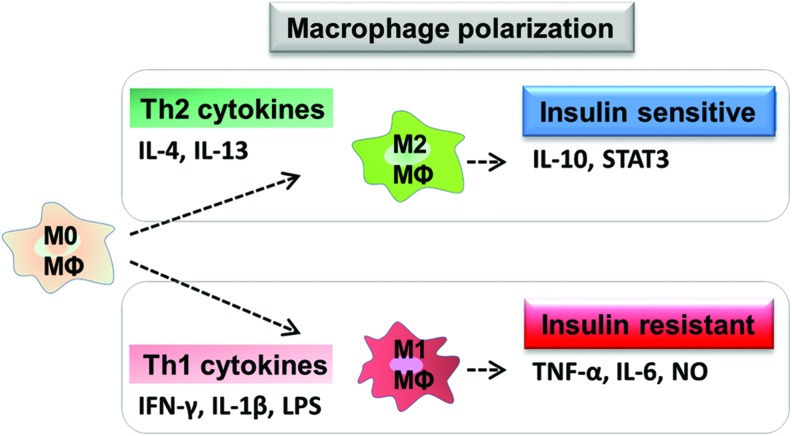
**Macrophage polarization and their effects on insulin regulation.** Th2 cytokines such as IL-4 and IL-13 differentiate quiescent (M0) macrophages to M2 phenotype. M2 macrophages maintain the insulin-sensitive state by the release of IL-10 and STAT3. In contrast, Th1 cytokines in the presence of LPS (or other TLR ligands) differentiate macrophages to M1 phenotype. M1 macrophages promote insulin resistance by the release of TNF-α, IL-6, and NO. STAT3, signal transducer and activator of transcription 3. To see this illustration in color, the reader is referred to the web version of this article at www.liebertpub.com/ars

The activation state of macrophages present in a tissue has a profound effect on disease biology. Macrophages are immune cells with a highly plastic phenotype ranging from highly proinflammatory to an anti-inflammatory phenotype, these activation states are induced when the cells are exposed to specific stimuli (Fig. 3). Macrophage phenotype has been studied for many years using the expression of key genes, cytokines, and cell surface molecules as hallmarks of macrophages existing at the polar extremes of phenotype [reviewed in Ref. (33, 66)]. The definition of macrophages as M1 or M2 is based on activation of cells *in vitro* with very defined stimuli, as defined by Mantovani *et al.* (64). M1 macrophages are generated after exposure of the cells to LPS, or FAs in the case of metabolic inflammation, which ligate TLRs on the cell surface, in the presence of interferon gamma (IFN-γ) (93). These cells have a broadly proinflammatory phenotype and upregulate both the cardinal proinflammatory cytokines TNF-α, IL-1, and IL-6, and characteristic genes such as nitric oxide synthase 2 (NOS2). These classically activated macrophages show enhanced killing of intracellular pathogens and low levels of the anti-inflammatory cytokine IL-10. When macrophages are exposed to IL-4 and/or IL-13, an alternatively activated M2 phenotype arises. These M2 macrophages secrete cytokines that oppose the M1-produced cytokines, including IL-10 and antagonists of the IL-1 pathway (IL-1ra and the decoy receptor IL-1RII), and express key marker genes such as increased arginase 1 (Arg-1), and Ym-1 [reviewed in Ref. (30)]. The cytokine IL-13 is known to induce the expression of M2 marker genes in both AT and liver (69). This M2 state of activation can be anti-inflammatory, but is also associated with allergy and immunity against pathogens. While *in vivo* such precise polarization of the cells is unlikely to happen, drawing parallels to the *in vitro* phenotype, defined by gene expression or cytokine/mediator production, allows some identification of macrophages seen in disease. Understanding the more varied stimuli that alter macrophage biology toward an M1 or M2-like phenotype and what mediates the positive or negative effect of these macrophages are key to our understanding of how macrophage biology plays a role in obesity and IR. For a comprehensive review of the paradigm of macrophage activation, see Mantovani *et al.* (64) and Martinez *et al.* (65).

Macrophages in lean AT are M2 phenotype, being F4/80^+^CD206^+^CD301^+^CD11c^−^ and sparsely distributed, whereas those in obese ATMs have an M1 phenotype, expressing F4/80^+^CD11c^+^ and form crown-like structures (CLSs) surrounding the adipocytes (22, 31) (Fig. 1). M1 macrophages mediate the metabolic complications of obesity, both in AT and by infiltration into other metabolic organs such as skeletal muscle (90). In mice (fed HFD), obesity induces ATM polarization from M2 to M1 phenotype (58). M1 macrophages (Th1) induce IR by producing inflammatory mediators, such as TNF-α, IL-6, and nitric oxide (NO) (58). M2 macrophages maintain insulin sensitivity by the anti-inflammatory actions of IL-10 and signal transducer and activator of transcription (STAT) 3 (58).

Recent studies in mouse models of obesity reveal some of the signaling pathways that mediate AT inflammation and the resulting macrophage polarization. JNKs are stress-activated protein kinases and are strongly associated with obesity and IR both in mice and humans. Saturated FAs, but not unsaturated FAs, activate JNK and inhibit insulin signaling *via* c-Src activation (39). Macrophage-specific deletion of JNK in mice protects from obesity-induced IR (fed HFD) by reducing infiltration of macrophages into pancreatic islets and ATM M1 polarization (35). Micro RNA-155 in adipose-derived microvesicles induced M1 macrophage polarization and caused chronic inflammation and local IR (111), indicating a further mechanism of obesity-induced proinflammatory signaling.

ATMs originate from bone marrow monocytes and infiltrate the tissue *via* blood circulation. MCP-1 stimulates the proliferation of ATM locally in VAT in genetically obese mice (ob/ob) (1). MCP-1 binds to chemokine (C-C motif) receptor 2 (CCR2) and causes macrophage migration (89). In obesity, the cytokine MIF is enhanced and linked to obesity-associated inflammation and IR, indicating that it may be a primary cytokine promoting ATM recruitment during obesity (24). MIF also promotes secretion of TNF-α, IL-6, and IL-1β, and inhibits IL-10 production, increasing inflammation (15). Obese (HFD-fed) MIF deficient mice were protected from weight gain and IR by attenuating M1 ATM infiltration, TNF-α, and increased expression of IL-10 (23). The STAT6 and nuclear receptors peroxisome proliferator-activated receptor (PPAR)-γ and PPAR-δ also play an important role in M2 macrophage polarization (11, 78). Macrophage-specific PPAR-δ deletion diminishes M2 macrophage activation, resulting in impaired glucose tolerance and systemic IR (77). PPAR-δ causes M2 macrophage polarization in both WAT and liver (69). IL-33 is recently identified as a member of IL-1 gene family and is expressed in human AT (adipocytes and preadipocytes) and also expressed in response to TNF-α (105). IL-33 induces the production of Th2 cytokines (IL-5, IL-13, and IL-10) and accumulation and polarization of M2 macrophages (CD206^+^) in WAT and is protective in obese mice by modulating AT inflammation (69).

Macrophages deficient in fatty acid synthase protect HFD-fed mice from IR, macrophage recruitment in AT, and chronic inflammation (102). NKT cells interact with CD1d, expressing antigen-presenting cells such as adipocytes, macrophages, dendritic cells, and B cells. In lipid excess environments, NKT cells interact with the endogenous lipid ligand CD1d on adipocytes and produce IFN-γ. This induces AT inflammation by increasing the expression of CD1d, MCP-1, and chemokine (C-X-C motif) ligand 16 (CXCL16) and decreasing expression of adiponectin (92). CD1d-deficient HFD-fed mice have decreased ATM recruitment, resulting in reduced obesity and improved insulin sensitivity (91).

Other immune cells such as neutrophils and T cells (subpopulations, CD4, CD8, and natural killer cells) are also increased in obese AT (Fig. 1). Neutrophils are reported to infiltrate the AT in 3 days after mice begin to consume a HFD (96). In lean AT, eosinophils are anti-inflammatory by secreting IL-4 and IL-13, but in obese AT, eosinophil content decreases. In obesity, regulatory T cells (T_reg_) decline and increased CD4^+^ and CD8^+^ T cells secrete proinflammatory cytokines. HFD feeding in mice causes infiltration of CD8^+^ T cells in WAT, which, in turn, activates the accumulation of F4/80^+^CD11b^+^ macrophages in WAT (73).

## The Role of Reactive Oxygen Species and Oxidative Stress, Macrophage Polarization

Reactive oxygen species (ROS) (oxygen-free radicals) are essential signaling molecules in various biological processes such as gene expression, protein translation, post-translational modification, and protein interactions. ROS such as superoxide anion (O_2_^−^), hydrogen peroxide (H_2_O_2_), and hydroxyl radical (^•^OH) are generated by the mitochondrial oxidative metabolism and degradation of FAs in peroxisomes, cytochrome P450 enzymes, Kupffer cells, and neutrophils [reviewed in Ref. (67)]. The mitochondrial respiratory system is a major contributor of ROS production in physiological conditions (72). Cells maintain the balance between oxidant and antioxidant defense molecules in addition to regulating their intracellular ROS levels. Over production of ROS may cause pathophysiological events such as oxidation of proteins and lipids and DNA damage, as a consequence cells repair damage, or die by necrosis or apoptosis (12).

Adipocytes synthesize ROS in the presence of glucose and palmitate. Excess amounts of glucose and palmitate increase the expression of ROS *via* nicotinamide adenine dinucleotide phosphate (NADPH) oxidase 4 (Nox4) in adipocytes (34). Treatment of macrophages with LPS and IFN-γ synthesizes NO production by increasing the production of inducible NOS (iNOS) [reviewed in Ref. (54)]. NO is known to inhibit mitochondrial respiration. HFD-fed iNOS-deficient mice are protected from infiltration of proinflammatory macrophages and AT fibrosis. *In vitro* macrophage-derived NO reduces the expression of mitochondrial biogenesis factors and increases hypoxia-inducible factor-1 (HIF-1α), DNA damage, and activation of p53 in preadipocytes (46). Macrophage-released NO activates p53 that results in suppression of PPAR-γ coactivator 1α (PGC-1α) and mitochondrial dysfunction in preadipocytes (46). Macrophages can additionally generate ROS *via* the activation of Nox2. ROS is produced in the presence of excess FAs; adipocytes increase oxidative stress *via* NADPH oxidase activation, which leads to dysregulated production of adiponectin, plasminogen activator inhibitor (PAI)-1, IL-6, and MCP-1 (28). Glucose-induced increases in ROS production in macrophages cause proinflammatory polarization and IR in mice [reviewed in Ref. (40)]. In obese mice, inhibition of NADPH oxidase leads to reduced ROS production in AT, diminished the dysregulation of adipokines, and improved diabetes and hyperlipidemia (28). HFD-fed and insulin-resistant obese mice showed increased production of ROS in adipocytes. Adipocytes in HFD-fed and obese insulin-resistant mice express elevated ROS production by the activation of PKC-δ and NADPH oxidase (95).

## Macrophage Polarization and Cellular Metabolism

**FIG. 4.**
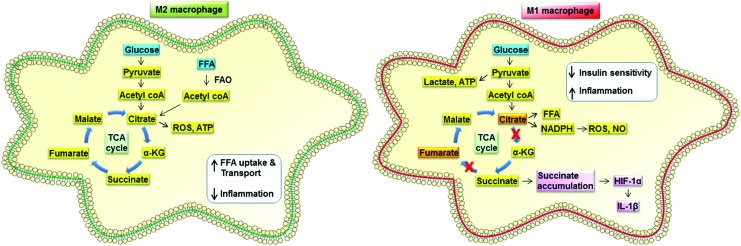
**Immunometabolic differences between M2 and M1 macrophages.** Both M1 and M2 macrophages can produce energy by glucose metabolism. M2 macrophages use OXPHOS and the TCA cycle, similarly to quiescent macrophages, but additionally demonstrate increased FFA uptake and oxidation (FAO) to supplement the TCA cycle and their energy needs. M1 macrophages increase glucose uptake and produce energy *via* oxidative glycolysis, resulting in lactate production. In addition, they exhibit a broken TCA cycle leading to the accumulation of both citrate and succinate. Succinate accumulation leads proinflammatory cytokine IL-1β release *via* HIF-1α. α-KG, alpha-ketoglutarate; FAO, fatty acid oxidation; HIF-1α, hypoxia-inducible factor-1α; NADPH, nicotinamide adenine dinucleotide phosphate; TCA, tricarboxylic acid. To see this illustration in color, the reader is referred to the web version of this article at www.liebertpub.com/ars

Alongside their well-characterized gene and protein expression signatures, M1 and M2 macrophages have markedly different metabolic processes. When in a resting state, macrophages meet their energy requirements using oxidative phosphorylation (OXPHOS). Glucose molecules enter glycolysis to produce pyruvate, with a single glucose molecule yielding two molecules of pyruvate. Under resting normoxic conditions, pyruvate enters the mitochondria where it is consumed by the tricarboxylic acid (TCA) cycle with ATP generated by OXPHOS. In contrast, macrophages activated under M1 conditions, such as after LPS stimulation, undergo dramatic metabolic changes. Uptake of glucose increases, and aerobic glycolysis becomes the principal metabolic pathway [reviewed in Ref. (54, 82)]. The increased glucose uptake increases flow through the glycolytic pathway, with increased pyruvate kinase muscle isozyme M2 (PKM2) expression, facilitating production of pyruvate to support lactate production and supply the TCA cycle (81). This altered metabolism, called the Warburg effect, causes buildup of ATP. The glycolytic intermediates support nucleotide and amino acid synthesis as well providing substrates for the pentose phosphate pathway to produce NADPH. The reduction in mitochondrial electron transport chain activity results in mitochondrial ROS production that, alongside the increased availability of NADPH for NADPH oxidase activity, results in a cell with a highly proinflammatory redox status (104). The Warburg effect is primarily associated with cells undergoing rapid proliferation, but this is not the case in M1 macrophages; instead, the increased glucose consumption is associated with rapid cytokine production and enhanced antimicrobial activity through ROS generation (104). Although a comparatively inefficient means to produce ATP, glycolysis is sufficient for macrophage function, providing glucose availability and uptake are not limiting. A combined metabolomics and transcriptomics approach has revealed that as well as a switch to glycolysis, M1 macrophages have a “broken” TCA cycle (48) (Fig. 4). The flow of metabolites around the TCA cycle is interrupted at the level of isocitrate dehydrogenase, leading to a reduction in the production of alpha-ketogluarate from citrate. This causes citrate to accumulate in M1-activated cells, which can then support FA synthesis. The “broken” TCA cycle also accumulates succinate in M1 macrophages, rather than proceeding to form fumarate and malate. The excess succinate production can lead to induction of IL-1β, adding to the proinflammatory nature of the M1 cells, and causes HIF-1α induction (81).

In contrast to M1 macrophages that synthesize FAs downstream of citrate accumulation, M2 macrophages metabolize FAs in addition to glucose to supply the TCA cycle (Fig. 4). The TCA cycle is intact in M2 macrophages and is coupled to OXPHOS (48). When differentiated using IL-4, M2 macrophages show a significant increase in FA uptake and oxidation (FAO or B oxidation) to fuel mitochondrial OXPHOS. Treatment of macrophages with either compounds to block FAO (etomoxir) or to inhibit OXPHOS (FCCP and oliogmycin) both decrease arginase activity, a defining feature of an M2 macrophage along with surface expression of CD301 and CD206 (42, 98). The induction of gene expression pathways supporting FAO and mitochondrial biogenesis by PPAR-γ and PPAR-γ coactivator 1-beta (PGC-1β) in response to IL-4 underpins the changes in metabolic state that occur with M2 differentiation (98). M2 macrophages use cell-intrinsic lysosomal lipolysis as a source for the FAs required by this metabolic program (42). Inhibiting lipolysis using tetrahydrolipistatin (orlistat) in M2 macrophages reduces OXPHOS, but has no effect on metabolism in M1 macrophages. Orlistat treatment reduces hallmark M2 cell surface marker expression, confirming the requirement of FAO for M2 differentiation (42). Uptake of FAs *via* the scavenger receptor (CD36) before lipolysis by lysosomal acid lipase appears to be an important mechanism providing substrate for FAO in M2 macrophages (42). Although providing convincing evidence that FAO is required for the M2 phenotype, nonpharmacological approaches have indicated that the link may be less straightforward. Conditional macrophage-specific knockout of carnitine palmitoyltransferase (CPT) 2, a mitochondrial protein essential for FAO, reveals that bone marrow-derived macrophages (BMDMs) can still express M2 markers in the absence of FAO (75). Whether FAO is an absolute requirement for M2 macrophage function or not, the association with this anti-inflammatory cell type is clear.

The strong link between macrophage polarization and cellular metabolism raises the possibility that modulation of macrophage metabolic state may be a therapeutic target. The glycolytic switch in macrophage metabolism as they undergo classical M1 activation is based on glycolysis being fundamental to the inflammatory capability of M1 macrophages, rather than a secondary result of M1 activation. Experiments to modulate expression of key elements of the metabolic pathways in macrophages have been undertaken in recent years to assess the extent to which macrophage inflammatory status can be modulated by targeting cellular metabolism. The glucose transporter (GLUT) 1 is upregulated in inflamed obese AT, colocalizing with macrophages in CLSs (27). GLUT1 overexpression in the RAW macrophage cell line is associated with increased glucose uptake, glycolytic rate, and lactate production. These cells exhibit enhanced gene expression and secretion of a range of inflammatory mediators, such as IL-1, IL-6, and TNF-α and, in the presence of LPS, further enhanced expression of the inflammatory mediator PAI-1 (27). These observations imply that altering metabolic status can control inflammation, in the absence of other inflammatory signals; however, a similar study by Nishizawa *et al.* (74) has reported conflicting findings. Overexpression of GLUT1 in the J774 cell line or in BMDMs produces the expected increase in glycolysis, but did not alter IL-6 expression (74). Similarly, *in vivo* experiments with retroviral GLUT1 overexpression in monocytes (driven by the CD68 promoter) after bone marrow chimerization did not demonstrate any altered cytokine secretion by peritoneal macrophages in the thiogylcollate model of peritonitis, despite increased glucose uptake measured using D-glucose positron emission tomography (18-FDG PET) analysis (74).

Macrophage phenotype can also be altered through altering FA uptake. Fatty acid transport protein 1 (FATP1) is reduced in M1 macrophages, but expression is maintained in M2 macrophages (50). Knockout of FATP1 in macrophages resulted in elevated energy production by OXPHOS both in M2 macrophages and resting or M1 activated cells. These changes were associated with a priming of macrophages toward the proinflammatory M1 state with a greater upregulation of NOS2 in M1-activated cells and a blunted expression of arginase 1 in M2-activated cells (50). *In vivo* experiments using bone marrow chimerization showed that loss of FATP1 in leukocytes alone increased weight gain, adiposity, and glucose intolerance under HFD conditions. AT in these mice showed signs of increased proinflammatory M1 macrophage activity with an increase in CLSs and proinflammatory gene expression (50). The ability of a single gene controlling FA metabolism in leukocytes to cause measureable changes in obesity and insulin sensitivity at the whole animal level underlines the potential power of altering immunometabolism. The converse experiment of overexpressing FATP1 in a macrophage cell line (RAW264.7) decreased GLUT1 expression using the M1 stimulus LPS and decreased glycolytic rate in both resting and stimulated cells. This change was accompanied by a decreased proinflammatory gene expression (50). Further evidence of an association of metabolic reprogramming with metabolic disease shows that obese and T2D individuals have reduced levels of FAO rates. The expression of CPT1A is higher in human ATM than mature adipocytes (63). CPT1 overexpression increases FAO levels in ATM. CPT1 expressing macrophages restored palmitate-induced increase in TG, inflammation, ER, and oxidative stress. Increase of FAO in lipid-treated adipocytes and macrophages reduced TG content, inflammatory cytokine levels, reduced ER stress, and ROS damage to macrophages, and improved insulin sensitivity in adipocytes (63).

These examples of the ability of the cell membrane nutrient transporters such as GLUT1 and FATP1 to alter macrophage phenotype indicate that local nutrient availability may also impact macrophage phenotype through the modulation of intracellular metabolism through excess or limited substrate availability. This seems likely to be of particular relevance in metabolic conditions typified by altered systemic glucose control and thus availability. As future studies investigate the immunometabolic state of macrophages in both lean and obese AT, the contribution of these metabolic alterations as potentially both causative and therapeutic will become clearer.

## Macrophage Metabolic Reprogramming

In addition to the examples of GLUT1 and FATP directly demonstrating modulation of macrophage metabolism, the identification of signaling pathways within macrophages that program the macrophage metabolic phenotype is a current research focus across multiple fields. We highlight here some of the recent findings that relate to relevant pathways associated with metabolic disease. Table 1 summarizes current targets that show promising relevance for future investigation in ATMs, which are covered in this review.

**Table 1.** Targets for Macrophage Metabolic Reprogramming in Adipose Tissue Macrophages

*Targets for macrophage metabolic reprogramming*	*M1 macrophages*	*M2 macrophages*	In vivo *effects relating to monocyte/macrophage function*	*Link with intracellular macrophage metabolism*	*Ref.*
GLUT1	Overexpression in RAW cells increases M1 cytokines: IL-1, IL-6, and TNF-α. An increase in IL-6 is not seen in J774 cells.	—	Endogenous expression is increased in inflamed obese adipose tissue.	Overexpression increases glucose uptake, glycolytic rate, and lactate production.	(27)
			Overexpression in monocytes increases glucose uptake but did not alter cytokine production.		(74)
FATP	Endogenous expression reduced on differentiation.	Endogenous expression maintained on differentiation.	Mice lacking FATP in leukocytes show increased weight gain, adiposity, and glucose tolerance on a high-fat diet.	Knockout of FATP increases energy production by OXPHOS in M1 and M2 macrophages.	(50)
	*FATP^−/−^* M1 macrophages show M1 priming, with increased iNOS expression.	*FATP^−/−^* M2 macrophages show blunted arginase expression		FATP overexpression decreases glycolytic rate in resting and M1 macrophages	
	FATP overexpression reduces GLUT1 expression.				
Notch	Notch1-RBP-J signaling promotes M1 polarization.	Notch-RBP-J signaling inhibits M2 macrophage polarization by downregulating JMJD3.	Notch inhibitor DAPT attenuated glycolysis, reducing glucose uptake and lactate formation in hepatic macrophages in response to alcoholic steatohepatitis.	Notch induces pyruvate dehydrogenase phosphatase that supports the TCA cycle.	(101)
	*Notch1^−/−^* hepatic macrophages show reduced M1 gene expression after LPS stimulation *in vitro*.			Notch regulates transcription of respiratory chain proteins.	(70)
					(108)
IL-10	IL-10 is induced by M1 macrophages and acts as a cell-autonomous regulator of glycolysis.	IL-10 is highly upregulated by M2 macrophages.	*IL-10^−/−^* macrophages from an inflammatory colitis model show a buildup of damaged mitochondria and increased inflammation because of inflammasome activation.	*IL-10^−/−^* M1 macrophages show increased glycolytic activity and reduced OXPHOS.	(45)
				IL-10 reduces GLUT1 expression on the cell surface.	(6)
CARKL	LPS downregulates endogenous CARKL expression.	M2 macrophages upregulate CARKL	—	CARKL is an orphan receptor in the pentose phosphate pathway.	(37)
	CARKL overexpression reduces IL-6 and TNF-α in response to LPS.			*CARKL^−/−^*macrophages show enhanced glycolysis, without additional stimulation.	
	*CARKL^−/−^* macrophages expression of M1 cytokines without further stimulation.				
IL-4	IL-4 treatment is not sufficient to repolarize M1 macrophages. Nitric oxide produced by M1 macrophages prevents repolarization.	IL-4 promotes M2 polarization. Repolarization to M2 requires mitochondrial function.	—	Inhibition of iNOS allows IL-4 to repolarize M1 cells, reducing glycolysis and promoting OXPHOS.	(97)
mTOR pathway	Inhibition of both mTORC1 and mTORC2 promotes M1 polarization.	*Tsc^−/−^* macrophages show defective M2 polarization in response to IL-4.	Macrophage-specific *mTORC1^−/−^* mice show less inflammation after high-fat feeding.	*Tsc^−/−^* macrophages show no increase in FAO in response to IL-4.	(49)
mTORC1/2 are multisubunit complexes within the pathway	Deletion of Tsc1 enhances mTORC1 activity. *Tsc1^−/−^* macrophages show increased proinflammatory cytokine production and reduced IL-10 production.		Global mTORC1 inhibition with rapamycin increases the number of M1 macrophages after high-fat feeding.	mTORC2-deficient macrophages show defective OXPHOS utilization after IL-4 stimulation.	(13)
					(83)

CARKL, carbohydrate kinase-like protein; FAO, fatty acid oxidation; FATP, fatty acid transport protein; GLUT-1, glucose transporter-1; IL-6, interleukin-6; iNOS, inducible NOS; JMJD3, Jumonji domain-containing 3; LPS, lipopolysaccharide; mTORC, mechanistic target of rapamycin complex; OXPHOS, oxidative phosphorylation; RBP-J, recombining binding protein suppressor of hairless; TCA, tricarboxylic acid; TNF-α, tumor necrosis factor alpha.

## Notch

**FIG. 5.**
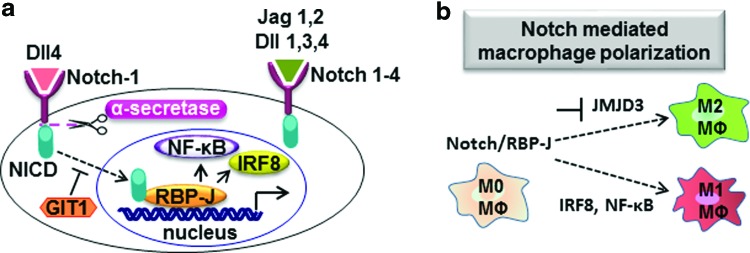
**(a) The role of Notch signaling in macrophage polarization**. Notch signaling promotes M1 macrophage polarization by synthesizing IRF8 and NF-κB and inhibits M2 macrophage polarization by downregulating JMJD3. **(b)** The Notch signaling pathway. The Notch ligands (Dll1, Dll3, and Dll4 and Jag1 and Jag2) bind to the Notch receptors (Notch 1–4). Upon ligand binding to the Notch receptor, proteolytic cleavage takes place (*via* α-secretase) in Notch receptor, resulting in the release of NICD. The NICD translocates to the nucleus and binds to RBP-J, resulting in the release of IRF8 and NF-κB. The GIT1 inhibits the Notch1-Dll4 mediated signaling. Dll, delta like; GIT1, G protein-coupled receptor-kinase interacting protein-1; IRF8, interferon regulatory factor 8; Jag, Jagged; JMJD3, Jumonji domain-containing 3; NF-κB, nuclear factor kappa light chain enhancer of activated B cells; NICD, Notch intracellular domain; RBP-J, recombining binding protein suppressor of hairless. To see this illustration in color, the reader is referred to the web version of this article at www.liebertpub.com/ars

The Notch signaling system has relevance to metabolic disease at multiple levels. The Notch signaling pathway consists of Notch receptors (Notch 1–4) and Notch ligands (delta-like [Dll]: Dll1, Dll3, and Dll4, and Jagged: Jag1 and Jag2), which are important for cell–cell communication, development, and required for cellular homeostasis [reviewed in Refs. (2, 55)]. Binding of Notch receptors to their ligands leads to proteolytic cleavage of Notch, resulting in the release of Notch intracellular domain (NICD) (Fig. 5a). The NICD binds to the recombining binding protein suppressor of hairless (RBP-J) in the nucleus [reviewed in Ref. (55)]. Notch-1 regulates the expression of Dll4 (Notch ligand) *via* RBP-J. The GPCR-kinase interacting protein-1 (GIT1) was proven to inhibit the Notch-1-Dll4 signaling (62). The Notch1-RBP-j signaling promotes M1 macrophage polarization *via* the synthesis of interferon-regulatory factor 8 and *via* NF-κB signaling and inhibits M2 macrophage polarization by downregulating Jumonji domain-containing 3 (70, 107) (Fig. 5b). In adipocytes, Notch signaling promotes production of proinflammatory cytokines (TNF-α, IL-1β) *via* NF-κB signaling, resulting in the infiltration of macrophages, low-grade systemic inflammation, and IR. In obesity, infiltrated macrophages activate Dll4 *via* NF-κB signaling (8). Notch-1 is necessary for the differentiation of 3T3-L1 fibroblasts into adipocytes by decreasing the expression of transcription factors PPAR-γ and PPAR-δ (29). Notch receptors and Notch targets are expressed highly in visceral epididymal WAT than in subcutaneous inguinal WAT. Inhibition of Notch signaling in obese mice (fed HFD) promotes browning of WAT, elevated expression of UCP1 in WAT, and ameliorates and transcription factor forkhead box protein O1 (FoxO1)-driven hepatic IR (9, 80). Moreover, inactivation of Notch signaling ameliorates obesity in obese mice (fed HFD) and reduces blood glucose levels (9). In hepatocytes, abnormal Notch signaling (gluconeogenesis and lipogenesis) leads to hyperglycemia and fatty liver disease. Notch regulates hepatic glucose production *via* the synergy of NICD and FoxO1 [reviewed in Ref. (8)].

In addition to these well-established roles for Notch proteins, this pathway has also been shown to have a direct effect on macrophage mitochondrial metabolism in a model of alcoholic steatohepatitis (ASH). Macrophages isolated from the liver of mice subjected to this model demonstrate the expression of M1 phenotypic markers such as iNOS, TNF-α, and IL-1β (108). *Notch1^−/−^* hepatic macrophages also showed an attenuation of M1 gene expression when challenged with LPS *in vitro*. Inhibition of Notch using the γ-secretase inhibitor DAPT (N-[N-(3,5-difluorophenacetyl)-L-alanyl]-S-phenylglycine *t*-butyl ester) attenuated the increase in glucose uptake and lactate production, hallmarks of increased glycolysis, in primary hepatic macrophages from ASH mice (108). Notch was shown to mediate effects on metabolism by induction of pyruvate dehydrogenase phosphatase, which has a key function in passing glucose to the TCA cycle. The ability of Notch to control macrophage metabolism was shown to be pleiotropic with additional transcription regulation of genes encoding respiratory chain proteins, encoded both by nuclear and mitochondrial DNA (108). Addressing the role of Notch in the biology of ATM in healthy and inflamed ATs has the potential to reveal a key element role for Notch in these macrophages as well.

## IL-10

We have discussed the two archetypal extremes of macrophage polarization in this review—M1 and M2 macrophages. A key cytokine produced by M2 macrophages is IL-10, which has an anti-inflammatory activity. A recent study has shown that IL-10 has the ability to directly affect macrophage metabolic reprogramming. Activation of BMDMs from *IL-10^−/−^* and control mice demonstrated that in response to LPS, IL-10-deficient cells demonstrated an exaggerated switch to glycolytic activity and reduction in OXPHOS (45). These effects could be reversed by rescue of the IL-10-deficient cultures with exogenous IL-10. IL-10 was shown to oppose metabolic switch by reducing macrophage glucose uptake as a result of opposing the cell surface upregulation of GLUT1 (45). When subjected to an inflammatory model, in this case a colitis model, macrophages *IL-10^−/−^* mice showed a buildup damaged mitochondria, which can contribute to exaggerated inflammation by activation of the inflammasome and production of IL-1β (45). This ability of IL-10 to control mitophagy indicates another facet of metabolic regulation by IL-10. The induction of IL-10 that occurs after LPS production has been proposed to be a cell-autonomous means of controlling macrophage glycolytic activity (6). While these studies have shown IL-10 produced by M1 macrophages has a role in modulating the metabolic reprogramming toward glycolysis, a question of interest is whether the paracrine production of IL-10 by M2 macrophages within a mixed population is also capable of modulating the metabolic activity of glycolytic M1 macrophages. Whether metabolic reprogramming occurs only within the cell itself, or whether it can be driven by the activation profile of other cells locally within tissue is an open question that has great impact on the therapeutic potential of these recent findings.

## Carbohydrate Kinase-Like Protein

The association of metabolism with macrophage phenotype is underlined by the ability of an unbiased screen for regulators of macrophage function to select a novel metabolic regulator. A kinase screen of 199 human kinase sequences in the RAW264.7 macrophage cell line to identify kinases altering TNF-α production identified carbohydrate kinase-like protein (CARKL) alongside known regulators such as p38 (37). Despite being screened for cytokine production, the gene identified is an orphan receptor in the pentose phosphate pathway. LPS treatment was shown to downregulate CARKL expression, but in the presence of plasmid-derived overexpression, production of “proinflammatory” cytokines including IL-6 and TNF-α was blunted, whereas expression of IL-10 was augmented (37). Knock down of CARKL produced changes within the macrophage cell line that mirrored LPS activation—namely the switch toward glycolysis; in the presence of LPS these cells demonstrated additional expression of TNF-α and IL-6 expression, indicating that CARKL loss is sufficient to at least, in part, mimic an M1-like macrophage phenotype (37). The regulation of CARKL by M1 and M2 stimuli is reciprocal as treatment of cells with M2 stimuli causes an upregulation of CARKL, indicating it has roles across multiple macrophage phenotypes, and interventions into CARKL function may have the ability to mediate macrophage metabolic reprogramming (37).

## IL-4

How far it is possible to reprogram individual macrophages versus the pool of macrophages present in the tissue as a whole is an important question when considering disease interventions. Do you need to replace the pool of cells present, or can the phenotype of the existing cells present be modulated. Certainly driving quiescent macrophages to either M1 or M2 phenotype is well described, but once committed to one of these phenotypes, how far can their phenotype now be modulated? In particular, can cells switch away from the M1-driven glycolytic metabolism once polarized? Rechallenging either mouse or human macrophages with IL-4 after previously polarizing them with M1 stimuli demonstrated that the IL-4 rechallenge did not cause the cells to express either marker genes (including *Mrc1* or *Fizz1*—mouse, *CCL22*, *CD206*, or *CCL24* human) or cell surface markers such as CD206 (mouse and human) (97). When cellular metabolism was assessed, the prior switching of macrophage metabolism to glycolysis and suppression of OXPHOS was not reversed when the cells were then restimulated with IL-4, indicating that they were metabolically trapped (97). The induction of iNOS and production of NO by M1 macrophages is a key feature of M1 macrophages; inhibition of NO production was shown to improve OXPHOS activity in M1 macrophages and to allow at least partial repolarization to an M2 phenotype as measured by cell surface marker expression (97). This study underlines, through the ability of iNOS modulation to alter macrophage metabolic and phenotypic polarization, that as we understand more about macrophage metabolism, the interplay of different cellular processes may unveil novel pathways for metabolic reprogramming.

## Mammalian Target of Rapamycin

The mammalian target of rapamycin (mTOR), a serine/threonine kinase, is involved in wide biological processes such as regulation of cell growth, proliferation, nutrient transport, and sensing environmental signals such as insulin, glucose, and amino acids (99). Macrophage-specific deletion of mechanistic target of rapamycin complex 1 (mTORC1) protects HFD-fed mice by reducing inflammation through inactivation of Akt and reducing IR by inhibiting the 1α/clun NH2-terminal kinase-nuclear factor kappa-light-chain-enhancer of activated B cells (IRE1α/JNK/NF-κB) pathway (49). Pharmacological inhibition of mTORC1 with rapamycin in HFD-fed mice worsened glucose intolerance and AT inflammation by increasing the number of polarized M1 macrophages, naive and activated cytotoxic T lymphocytes, and proinflammatory markers such as TNF-α, IL-6, and MCP-1. Moreover, inhibition of mTORC1 and mTORC2 with torin 1 promoted M1 macrophage polarization in BMDMs (83). The tuberous sclerosis complex comprising 1 (Tsc1) deficiency in BMDMs exhibits increase in production of proinflammatory cytokines, activates mTORC1, and reduces IL-10 expression. Moreover, BMDMs deficient in Tsc1 exhibit decrease in M2 macrophage markers (Arg-1, Fizz1, macrophage galactose-type lectin-1 [Mgl1/CD301] and 2, Ym-1, and PGC-1β). Tsc1-deficient BMDMs treated with IL-4 exhibit mTORC1-mediated decrease in Akt activation that contributes to defect in M2 macrophage polarization (13, 83). These strong impacts on macrophage polarization imply that immunometabolism may be altered by targeting the mTOR pathway and indeed Tsc-1-deficient BMDMs show no increase in FAO in response to IL-4 stimulation (13). The seemingly contradictory finding that both inhibition of mTORC1-promoting M1 differentiation and activation of mTORC1, by Tsc1 deletion, decreasing M2 polarization, indicates that there is more we need to understand about regulation of this pathway under both physiological and pathophysiological conditions. The mTORC2 complex is required for M2 macrophage polarization, with loss of the defining complex subunit Rictor preventing increased OXPHOS utilization after IL-4 stimulation and reducing expression of characteristic M2 genes (43). These new links of the mTOR pathway to macrophage metabolism, given the existing association of this pathway with AT biology, make this a very relevant pathway for study in ATMs.

## Future Questions and Challenges

The striking changes in macrophage metabolism that occur with differential polarization have opened up an exciting new area for research in metabolic diseases. We have highlighted here some of the key mediators within the cell that act as modulators of macrophage metabolic function. Much of the fundamental analysis of macrophage metabolism has been performed in mouse models and *in vitro* derived or activated cells, which allows development of a defined system of homogenously polarized cells. However, *in vivo* macrophage phenotypes are rarely so clear. Defining the metabolic profile of primary macrophages isolated from *in vivo* tissues is a crucial next step for this field. Although AT and ATM show elements of an M2 to M1 switch with obesity, the precise phenotype of these cells will be unique to their local environment. For example, ATM populations have been shown to contain lysosome-rich and lipid-laden cells (109). These ATM features, and other phenotypic changes caused by obesity, diabetes, and other metabolic conditions, are likely to be manifest in their metabolic profile, as it is their cytokine output and gene expression.

As we begin to understand how far macrophage metabolism may define the phenotype of activated cells, it will become apparent how far other cellular functions that may be beneficial or drive disease can be altered by targeting cellular metabolism. Can we target macrophage metabolism to within AT to modulate their inflammatory phenotype in obese or diabetic individuals? Is there scope to fundamentally alter the phenotype of the cells to an anti-inflammatory phenotype that might actively contribute to resolving disease, rather than just reducing inflammatory signaling? Is immunometabolism a target for AT “hyperglycemic memory,” whereby previous IR or diabetes confers long-term adverse effects on metabolism and cardiovascular risk, even when glycemic control is achieved and maintained? Whereas epigenetic changes related to diabetic complications in “end organ” cells (*e.g.,* renal, endothelial, neuronal, and smooth muscle cells) are well described, the importance of “metabolic memory” in immune cells in AT will be important to investigate. Some initial experimental evidence already indicates an important role for epigenetic regulation of macrophage differentiation. In ATM of obese (ob/ob) mice, expression of the DNA methyltransferases DNMT1 is reduced in M2 macrophages compared with that in M1 macrophages. Inhibition of DNMT1 in ob/ob mice, either by pharmacological agent 5-aza-2′-deoxycytidine or by myeloid-specific deletion of DNMT1, promotes M2 macrophage differentiation by decreasing the PPAR-γ1 promoter DNA methylation (101). But are genes that encode or regulate macrophage metabolism targets for epigenetic modification?

The ability of cell surface nutrient uptake receptors such as GLUT1 to alter macrophage metabolism hints that nutrient availability locally, and potentially systemically in the case of metabolic disease, may alter macrophage phenotype. This could reveal a mechanism by which interventions not directly targeting macrophages may in fact have potent effects on macrophage metabolism. For example, interventions such as exercise training could have important effects on AT inflammation, beyond weight loss. For example, in obese mice, exercise training reduced macrophage clusters in AT and increased the number of CD8^+^ T cells (53). Exercise training in HFD-fed mice also inhibits AT inflammation by inhibiting TNF-α, TLR4, the number of F4/80 macrophages, and CD11c^+^ cells (an M1 macrophage marker), whereas CD163^+^ cells (an M2 macrophage marker) are decreased (60). This suggests that exercise training might induce a phenotypic switch of M1 macrophages to M2 macrophages in AT (47, 52). Revisiting these findings now we understand that the importance of macrophage metabolic reprogramming would provide insight into whether existing interventions to combat obesity are altering AT biology at the level macrophage metabolism. As our knowledge of macrophage metabolic programming in AT builds, there will be increasing scope for targeting this aspect of macrophage biology as a therapeutic strategy in metabolic diseases.

## Abbreviations Used

ASH: alcoholic steatohepatitis
AT: adipose tissue
ATMs: adipose tissue macrophages
BAT: brown adipose tissue
BMDMs: bone marrow-derived macrophages
BMI: body mass index
CARKL: carbohydrate kinase-like protein
CLSs: crown-like structures
CPT: carnitine palmitoyltransferase
FA: fatty acid
FAO: fatty acid oxidation
FATP1: fatty acid transport protein 1
FFA: free fatty acid
FFAR: free fatty acid receptor
FoxO1: forkhead box protein O1
GLUT: glucose transporter
GPCRs: G protein-coupled receptors
HFD: high-fat diet
HIF-1α: hypoxia-inducible factor-1
IFN-γ: interferon gamma
IL-6: interleukin-6
iNOS: inducible NOS
IR: insulin resistance
IRS-1: insulin receptor substrate-1
JMJD3: Jumonji domain-containing 3
JNK: c-Jun NH2-terminal kinase
LPS: lipopolysaccharide
LTs: leukotrienes
MCP-1: monocyte chemotactic protein-1
MIF: macrophage migration inhibitory factor
mTOR: mammalian target of rapamycin
mTORC1: mechanistic target of rapamycin complex 1
NADPH: nicotinamide adenine dinucleotide phosphate
NF-κB: nuclear factor-kappaB
NICD: Notch intracellular domain
NO: nitric oxide
NOS2: nitric oxide synthase 2
PAI: plasminogen activator inhibitor
PGC-1β: PPAR-γ coactivator 1-beta
PI3-k: phosphatidylinositol 3-kinase
PKC-δ: protein kinase C-delta
PPAR: peroxisome proliferator-activated receptor
PTP1B: protein-tyrosine phosphatase 1
RBP-J: recombining binding protein suppressor of hairless
ROS: reactive oxygen species
Sfrp5: secreted frizzled-related protein 5
STAT: signal transducer and activator of transcription
T2D: type 2 diabetes
TCA: tricarboxylic acid
TG: triglyceride
TLR4: toll-like receptor 4
TNF-α: tumor necrosis factor alpha
Tsc1: tuberous sclerosis complex comprising 1
UCP1: uncoupling protein 1
VAT: visceral AT
WAT: white adipose tissue
